# Advances in Aptamer-Based Biosensors for the Detection of Foodborne Mycotoxins

**DOI:** 10.3390/molecules29163974

**Published:** 2024-08-22

**Authors:** Yangyang Li, Dan Zhang, Xiaoyuan Zeng, Cheng Liu, Yan Wu, Cuicui Fu

**Affiliations:** 1 Chongqing Key Laboratory for New Chemical Materials of Shale Gas, College of Chemistry and Chemical Engineering, Yangtze Normal University, Fuling, Chongqing 408100, China; 20170201@yznu.edu.cn (Y.L.); 18388441102@163.com (X.Z.); 17723164432@163.com (C.L.); wyan2018@163.com (Y.W.); 2 School of Cable Engineering, Henan Institute of Technology, Xinxiang 453003, China; danzhangimnu@163.com

**Keywords:** aptamer, dual-mode biosensor, foodborne mycotoxins

## Abstract

Foodborne mycotoxins (FBMTs) are toxins produced by food itself or during processing and transportation that pose an enormous threat to public health security. However, traditional instrumental and chemical methods for detecting toxins have shortcomings, such as high operational difficulty, time consumption, and high cost, that limit their large-scale applications. In recent years, aptamer-based biosensors have become a new tool for food safety risk assessment and monitoring due to their high affinity, good specificity, and fast response. In this review, we focus on the progress of single-mode and dual-mode aptasensors in basic research and device applications over recent years. Furthermore, we also point out some problems in the current detection strategies, with the aim of stimulating future toxin detection systems for a transition toward ease of operation and rapid detection.

## 1. Introduction

FBMTs entering the human body through ingestion to a certain extent can cause the occurrence of diseases. To date, various FBMTs have been identified and classified, mainly according to toxicology, as ochratoxins (OTs), fumatoxins (FBs), aflatoxins (AFs), zearalenone (ZEN), deoxynivalenol (DON), and T-2 toxin [1,2,3,4]. These toxins have certain thermal stability and can accumulate in the food chain, potentially causing lesions in organs such as the liver and kidney at an extremely low concentration [5,6,7,8]. Therefore, it is absolutely crucial to develop toxin detection methods that are highly sensitive, straightforward to operate, and cost-effective, as they will greatly contribute to the health and well-being of both humans and animals.

In recent decades, various forms of detection methods for FBMTs have been developed that contribute greatly to public health. Thin layer chromatography (TLC), as an early chemical analysis method, is suitable for primary large-scale sample screening due to its stability, simple operation, and low cost [9]. Currently, advanced TLC scanners have effectively promoted their applications in qualitative and quantitative detections [10,11]. However, this instrument analysis method, similar to gas chromatography [12], high performance liquid chromatography [13], mass chromatography spectrometry [14], atomic absorption spectroscopy [15], X-ray fluorescence spectroscopy [16], etc., typically requires expensive instrument equipment, skilled operators, highly toxic chemical reagents, and cumbersome pretreatment processes, which consume a lot of time and labor and ultimately limit its functionality to some extent. Furthermore, the use of antibodies as recognition elements, coupled with various signal transduction mechanisms in immunoassays, has received considerable attention for their precise capture and significant detection of diverse targets in numerous fields [17,18,19,20,21]. As the most effective immunoassay method, the enzyme-linked immunosorbent assay has the advantages of selectivity, sensitivity, and autonomy [22]. However, there are still some drawbacks, such as an expensive detector, a long test period, and the reliance on a professional operator. For this reason, biosensors based on specific probes that can accurately and quickly capture target toxins have become an ideal candidate [23,24].

**Scheme 1:**
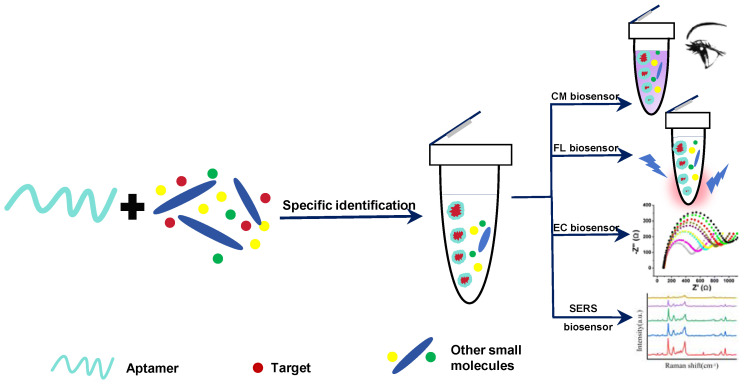
Different detection modes of aptamer-based biosensors.

Aptamers, also known as “chemical antibodies”, are oligonucleotide sequences that can be selected by the systematic evolution of ligands by exponential enrichment (SELEX). They have high affinity and specificity for various biological ligands, exhibiting unique biological and chemical properties [25]. Compared to natural antibodies, aptamers have the following advantages [26]: First, the aptamer has a higher surface density, which helps to improve the efficiency in binding of its target [27]. Second, aptamers have higher stability, making the conditions for storage less stringent than those for the storage of natural antibodies [28]. Third, aptamers can be customized to meet specific requirements, including synthesis, production, and purification [29]. Overall, as an oligonucleotide sequence, aptamers have a highly flexible structure and can be modified through appropriate post-SELEX techniques [29]. Significantly, considering the aptamer’s remarkable stability and resistance to degradation, its performance will remain unaffected following functional group modifications. Moreover, the aptamers, which are both cost-effective and non-toxic, are selected in vitro without the need for animal experiments. [30]. Based on the above advantages, aptamers can specifically bind to a variety of target substances, such as metal ions [31], amino acids [32], nucleotides [33], small organic molecules [34], toxins [35], enzymes [36], cells and bacteria [37,38], and other proteins [39]. Aptasensors based on aptamer and target specific recognition can be combined with chemical materials to convert detection results into different signals, such as chromaticity (CM) [40], fluorescence (FL) [41], electrochemistry (EC) [42], photoelectrochemistry (PEC) [43], surface enhanced Raman spectroscopy (SERS) [44], etc., to achieve qualitative or quantitative detection of targets (Scheme 1). As a result, the single-mode aptasensors capable of outputting a single signal and the dual-mode aptasensors capable of simultaneously outputting two signals have received widespread attention. Among them, the dual-mode aptasensor can verify two signals simultaneously, thereby expanding the detection range and enhancing overall accuracy. In recent years, various forms of dual-mode aptasensors have been frequently reported for the analysis and detection of biological targets. For example, Liu et al. reviewed the latest progress of SERS dual-mode aptasensing technology in the detection of pathogenic bacteria, biotoxins, and drug residues [45]. In addition, as the concern with food safety rises ceaselessly, dual- and multi-mode aptamer sensing technology will become an important research topic.

Aptamers are considered superior molecular recognizers, and aptamer-based biometric techniques have the potential to replace traditional methods, offering quicker, more sensitive, and highly reliable detection. In this review, we have focused on the recent development and future challenges of dual-mode aptamer sensors. It is expected to provide valuable information for the detection of FBMTs.

## 2. Detection Strategies on Aptamer Sensors for Foodborne Mycotoxins

### 2.1. Single-Mode Aptamer Sensors

On the basis of the specific binding of aptamer and target, the concentration of target substance can be converted into measurable physical signals by appropriate techniques. For this reason, aptamer sensors with different detection modes and suitable for different detection objects have been developed in recent years.

**Figure 1:**
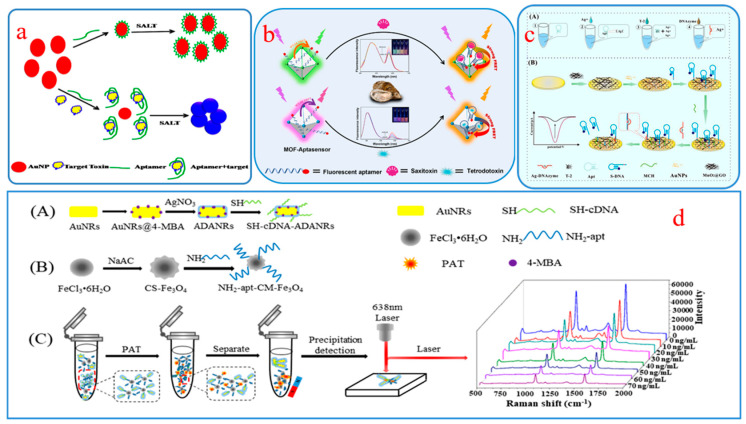
(**a**) AuNP-based CM detection assay format; (**b**) Sensing mechanism toward STX and TTX based on NMOFs-aptasensor; (**c**) Sensing process in microtubule (**A**) and on electrode surface (**B**); (**d**) Preparation for SH-cDNA-ADANRs (**A**) and NH_2_-apt-CS-Fe_3_O_4_ (**B**), and SERS detection schematic (**C**).

CM aptamer sensors have attracted scientists’ attention for their fast detection speed, the ability to be seen with the naked eye, low equipment price, and portability. The detection purposes are achieved based on color change of the target substance [46], change of cationic polymers [47], oxidation of substrate by enzyme [48], catalyzed oxidation of nanomaterials [49], or the aggregation or disaggregation of nanoparticles [50,51]. Typical CM aptamer sensors often employ a sandwich or hairpin structure to identify and capture targets [52,53,54]. Bhairab et al. developed a sandwich structure CM aptasensor using gold nanoparticle (AuNP) as a CM probe to detect enterotoxin B (SEB). When SEB exists, the color change of the system can be observed with the naked eye, and the concentration of SEB can be measured by instruments. The results show that the linear concentration response range of SEB was 50 μg/mL to 0.5 ng/mL (Figure 1a) [50].

The FL aptasensor realizes quantitative detection through fluorescence change via the combination of aptamers and a fluorescent probe, which is more sensitive and selective in comparison with colorimetry. The sandwich structure applied to CM sensors can also be used in FL sensors [55,56,57,58]. Dou et al. constructed a biosensor for tetrodotoxin (TTX) sensing by combining with zirconium fluorescent nanoscale metal-organic frameworks (NMOFs) and TAMRA labeled aptamer. In the process of identifying and capturing TTX, the structure of the aptamer will undergo a change, resulting in a decrease in the green light from NMOFs, while the red emission of the TAMRA labeled aptamer will increase due to the fluorescence resonance energy transfer (FRET) effect. This strategy has been successfully used for toxin sensing in shellfish samples, with a limit of detection (LOD) of 3.07 nM for TTX (Figure 1b) [59].

In 2004, Ikebukuro et al. developed an aptamer biosensor with current as a detection signal for the first time to quantitatively analyze thrombin [60]. After 20 years of evolutionary development, EC aptamer sensing technology has made great progress in modifying the electrode surface [61] and developing multi-signal output modes [62] so as to adapt to the detection of different labeled substances. In one study, an EC aptasensor was designed for quantitative analysis of the T-2 toxin based on the amplification of the signal assisted by Ag^+^-dependent DNAzymes. In the presence of T-2 toxin, Ag^+^ that has already been captured by the aptamer will be released, ultimately leading to a decrease in the EC signal. The results of detecting T-2 toxin in beer indicate that this method exhibits enormous potential in food safety and quality control (Figure 1c) [63].

Raman spectroscopy can obtain information on the chemical structure of substances at a molecular level and provide fingerprint recognition spectra [64]. At present, SERS technology developed on the basis of Raman scattering can significantly enhance Raman signals by generating electromagnetic and chemical enhancements on the substrate, thereby improving the detection sensitivity of targets [65,66,67]. Combining SERS technology with aptamer recognition ability and utilizing the advantages of fingerprint information, rapid detection, and ultra-high sensitivity, SERS aptamer sensors have been successfully applied to detect various FBMTs [68,69]. Guo et al. designed a SERS aptamer sensor by combining Au-Ag composite (ADANR) with chitosan-modified Fe_3_O_4_ NP (CS-Fe_3_O_4_) [70]. In this sensing system, the ADANR was a signal probe, while the CS-Fe_3_O_4_ with aptamer modification served as the capture probe (Figure 1d). It has an LOD of 0.0384 ng/mL for detecting Patulin (PAT) in actual samples, with a recovery range of 96.3% -108%, demonstrating good sensitivity and specificity.

In addition to the commonly used aptasensors mentioned above, the combination of aptamers with other cutting-edge technologies also has potential in detecting mycotoxins, including PEC biosensors [71], acoustic biosensors [72], magneto-impedance biosensors [73], thermometric biosensors [74], etc. PEC sensing technology incorporates photoelectric conversion properties of substances, creating a powerful analytical tool known as the PEC biosensor. The sensor exhibits exceptional sensitivity and remarkably low background signals, making it an invaluable asset in various analytical applications [71]. Acoustic sensors achieve precise targeting analysis by relying on the relevant physical properties of sound waves, namely changes in sound velocity, resonance frequency, and dissipation, which are directly related to the quantity of the targeted objects. The most notable feature of acoustic sensors is their exceptional sensitivity, which has garnered significant attention [72]. Magneto-impedance sensors generate signals based on the changes in electrical resistance of magnetic particles due to an applied magnetic field. These magnetically responsive particles, once modified, can bind to a variety of targets such as enzymes, bacteria, mycotoxins, proteins, etc., making them highly versatile and promising for use in a wide range of biomedical applications [75]. Thermometric sensors use temperature changes caused by biochemical reactions as their basis. The key component of these sensors is a biological receptor adhered to a thermistor. After the bioreceptor undergoes a biochemical reaction with its target, the resulting change in temperature will alter the thermistor’s resistance. The magnitude of this temperature change is directly proportional to the amount of the target, making it possible to achieve quantitative analysis [74].

### 2.2. Dual-Mode Aptamer Sensors

A dual-mode aptamer sensor can achieve dual-signal output through a multifunctional probe. Commonly used dual-mode probes include integrated probes and combined probes. The former usually requires a molecule with dual-signal response function, which can be easily modified to achieve dual-mode detection, but its quantity and types are often limited, whereas the latter can freely choose and combine probes according to demand to catalyze the generation of different signals from two substrates, resulting in the combined dual-mode probes being more applicable and practical. Compared with single-mode aptamer sensors, dual-mode aptamer sensors have received more widespread attention. We summarize some representative information on these two types of sensors for toxin detection and list them in Table 1.

#### 2.2.1. CM-FL Aptamer Sensors

The most prominent feature of the dual-mode CM-FL sensing system is its ability to simultaneously visualize detection and imaging. Furthermore, the difference in sensitivity between the CM and FL probes enables the dual-mode aptasensors of the CM-FL to satisfy the requirements for both qualitative analysis and quantitative detection.

**Figure 2:**
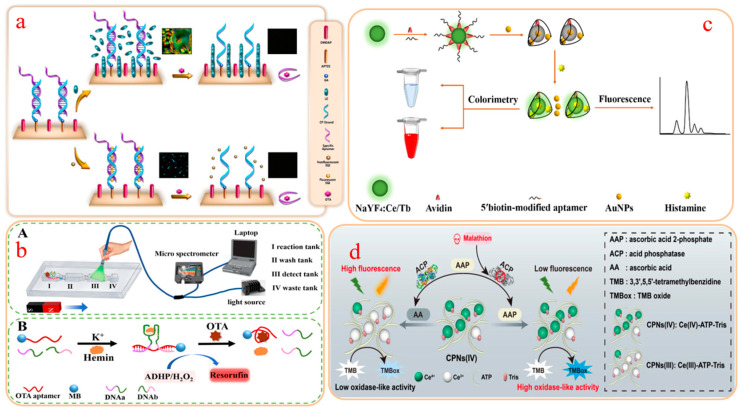
(**a**) Unlabeled CM-FL aptasensor for detection of OTA; (**b**) The portable CM-FL aptamer sensor device (**A**) and the detection principle of OTA (**B**); (**c**) Fluorometric and CM aptasensor for detection of histamine; (**d**) CPNs(IV)-based aptasensor for malathion detection.

Khalil et al. designed an unlabeled dual-mode CM-FL aptamer sensor for ultrasensitive detection of OTA (Figure 2a) [84]. The functional basis of the sensor was the arrangement rule of the liquid crystal (LC) and the connection mode between SGI dye and double-stranded DNA structure. The specific binding between OTA and the aptamer leads to the destruction of the DNA double-stranded structure, and the orientation state of the LC molecules also changes. These changes can be converted into optical image signals and observed through a polarized light microscope. On the other hand, OTA can also affect the number of SGI dye and observe the fluorescence brightness trend through fluorescence microscopy. Based on colorimetric and fluorescence responses, the aptasensor has detected ultralow levels for OTA with LODs of 0.047 aM and 34 fM, respectively, demonstrating good sensitivity and specificity. Luo et al. constructed a CM-FL aptamer sensing platform based on the coumarin benzothiazole molecule (DR1) and OTA binding aptamer for quantitative analysis of OTA [85]. Through OTA competition, this sensor generated fluorescent signals to provide a fast, label-free, and sensitive detection solution. In CM mode, the presence or absence of OTA will affect the color conversion of DR1, from purple to blue or from blue back to purple. Based on these optical conversion features, they have established a smartphone-based detection system that can convert color changes into RGB values, achieving visualization of OTA detection in real samples. The detection object is still OTA, and in a recent study, a portable CM-FL aptamer sensor based on the G-quadruplex DNAzyme has been designed (Figure 2b) [86]. The G-quadruplex DNAzyme probe was self-assembled from OTA aptamers coupled with magnetic beads and two fragments of DNA and hemin, used to catalyze Amplex Red and generate strong fluorescence signals. However, in the presence of OTA, the structure of the probe was disrupted, leading to a decrease in catalytic activity and showing quantitative detection of OTA. The results show that the LOD for CM and FL are 0.008 μg/kg and 0.011 μg/kg, respectively, and the recovery of the wheat sample is in the range of 76.0–82.5%.

Based on aptamer@NaYF_4_:Ce/Tb and AuNPs, Huang et al. developed a simple and rapid CM-FL sensing platform to detect histamine in fish [95]. The NaYF_4_: Ce/Tb as donor transfers energy to AuNP, which leads to the quenching of FL signals in the detection system. However, when histamines are present, AuNP-captured aptamers will be released from their surface, inducing fluorescence recovery (Figure 2c). This dual-signal-responsive aptasensor exhibits good detection sensitivity with an LOD of 4.57 nmol/L. The sensing platform has been successfully applied to the detection of histamine in fish, with recovery of 82.19–105.94%.

In the detection of food pesticide residues, Niu et al. designed a dual-mode aptasensor based on CPNs (IV) that can provide two independent and non-interference CM and FL signals. The sensing principle was the target-induced acid phosphatase inhibitory activity and its potential impacts on the luminescence and oxidase mimetic activity of CPNs (IV) (Figure 2d). In the malathion detection experiment, this sensing platform demonstrated good analytical performance [96].

#### 2.2.2. SERS-FL Aptamer Sensors

SERS technology can significantly enhance Raman signals by exposing the analyte to the substrate and generating hotspots. Combining SERS detection technology with aptamers can further enhance the strength of SERS signals and improve detection accuracy and sensitivity [97]. The FL aptamer sensors obtain signals on the basis of FL intensity of direct or indirect chemical reactions between the analyte and substrate, leveraging the high detection sensitivity of FL and the high specificity of aptamer.

**Figure 3:**
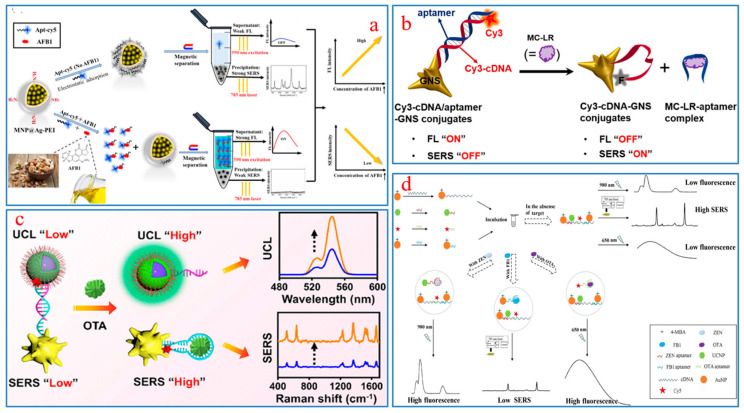
(**a**) Dual-signal output mechanism of SERS-FL aptasensor for determination of AFB1; (**b**) Working principle of the on-off-type aptasensor; (**c**) The “turn on” UCL-SERS aptasensor for detection of OTA; (**d**) Sensing mechanism of trimer-based high-throughput sensors.

In recent years, SERS-FL aptamer sensors, which combine the advantages of SERS, FL, and aptamer, have shown great potential in the detection and analysis of FBMTs. Sun et al. constructed a SERS-FL dual-mode aptamer sensor using the fluorescent dye cy5 as an FL and Raman dual probe. In addition, silver coated polyethylene imine-modified magnetic nanospheres were used to absorb the cy5-modified aptamer [87]. The sensor detected AFB1 in peanuts, walnuts, and almonds, and the results showed that the linear ranges of FL and SERS methods were 0.2–20,000 ng/mL and 0.001–1000 ng/mL, respectively, with LODs of 0.135 ng/mL and 0.45 pg/mL, respectively (Figure 3a). The sensors that integrated SERS and FL technologies demonstrated excellent complementarity in terms of detection range and LOD.

Precious metals with rough surfaces, such as gold and silver, often play an important role in SERS technology [89,98]. In one study, a SERS-FL dual detection signal aptasensor was designed by the high affinity of Cy3cDNA chains for plasma gold nanostars (GNSs) for sensitive detection of hepatotoxin MC-LR [99]. The presence or absence of MC-LR will cause signal changes in FL “off” and SERS “on” or FL “on” and SERS “off” to achieve dual-mode quantitative detection of the target substance (Figure 3b).

Upconversion nanoparticles (UCNPs) have advantages such as good chemical stability, sharp emission bands, a high signal-to-noise ratio, and low photobleaching and toxicity, and their anti-Stokes properties prevent coexcitation between donors and receptors, thereby reducing the possibility of false positive signals [100]. Therefore, UCNP is considered an excellent probe for FL biosensor [101]. As in Figure 3c, Wu et al. designed a “turn-on” SERS-FL dual-signal sensing platform by hybridizing complementary DNA coupled UCNPs and organic dye TAMRA-aptamer-modified AuNPs [88]. In the quantitative detection of OTA, simultaneous enhancement of SERS and FL signals was achieved because of the preferential binding between the aptamer and OTA and the conformational changes of the aptamer. The results showed that the LOD of SERS and FL were 8.6 pg/mL and 3.2 pg/mL, respectively. In addition, its recoveries of SERS and FL were 92.4–108% and 95.2–103%, respectively, for the detection of OTA in beer.

The SERS-FL dual-mode aptamer sensor also plays an important role in detecting multiple FBMTs simultaneously. In a study, an aptasensor based on SERS and FRET technology was assembled for simultaneous determination of three toxins; that is, OTA, ZEN, and FB1 [102]. The sensing principle is shown in Figure 3d. The LODs for OTA, ZEN, and FB1 are 0.01 ng/mL, 0.03 ng/mL, and 0.02 pg/mL, respectively, which demonstrate good selectivity and sensitivity and provide useful information for the development in the detection of multiplexed FBMTs.

#### 2.2.3. EC-PEC Aptamer Sensors

Among various biosensing technologies, EC-PEC dual-mode aptamer sensors have always been a research hotspot due to their fast response speed, simple preparation, good portability, and high sensitivity [103,104,105]. The technical core of EC-PEC dual-mode aptamer sensors to achieve high quality detection is the selection of appropriate semiconductor materials and photoactive materials to overcome the recombination of photogenerated carriers [106].

**Figure 4:**
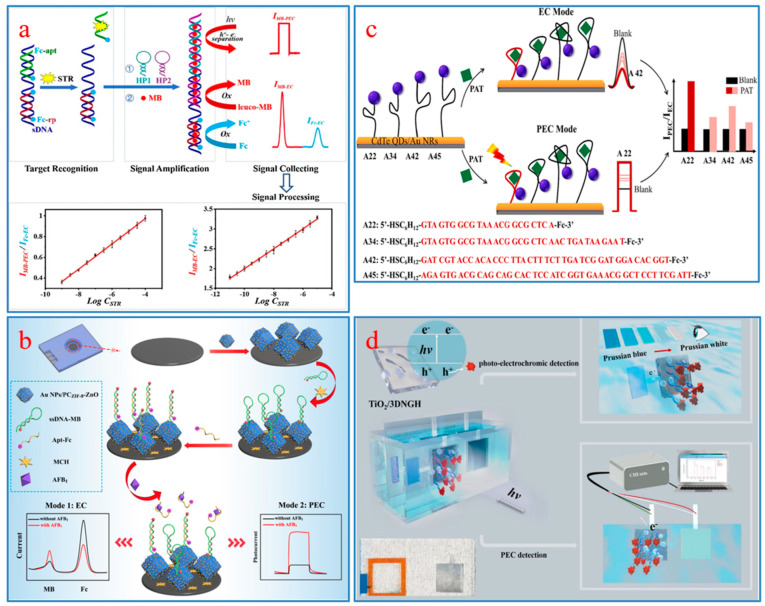
(**a**) Working principle of the dual-ratiometric aptasensor based on EC and PEC assays; (**b**) The detection of AFB1 in EC mode and PEC mode; (**c**) Priority binding order of the four aptamers with PAT; (**d**) Mechanism diagram of PEC/visualization dual-mode detection of DON.

The Yan research group designed an in situ coupling aptasensor for EC and PEC using methylene blue (MB) as a bifunctional probe [107]. Among them, MB serves as a bridge for the coupling of EC and PEC and simultaneously outputs two types of signals (Figure 4a). In the detection of streptomycin (STR), the linear ranges for EC and PEC are 1 × 10^−11^–1 × 10^−5^ M and 1 × 10^−9^–1 × 10^−4^ M, respectively. Zhang et al. used AuNP/PCZIF-8-ZnO composite material as a photoelectron substrate and assembled a paper-based EC-PEC aptasensor based on the distance modulation sensing strategy for quantitative analysis of AFB1 [91]. AuNP/PCZIF-8-ZnO with good conductivity can accelerate the electron migration rate and improve photoelectric conversion efficiency. Furthermore, the target substance AFB1 plays a role in the regulation of the distance between electroactive molecules and AuNP/PCZIF-8-ZnO, leading to changes in EC and PEC signals (Figure 4b). The dual-mode detection device has a wide detection range and a low detection limit, and it has shown good potential in the detection of AFB1 in actual samples.

Specific binding of different aptamers to the same target may result in significant differences in sensing effects. For example, Liu et al. selected different sequences of aptamers (i.e., A22, A34, A42, A45) to design four EC-PEC dual-mode sensors and compared their performance differences in the detection of PAT [92]. They found that although A42 showed the highest affinity for PAT, when the synergistic effects of steric hindrance and electron transfer distance were considered, A22 became the best choice (Figure 4c). This study explored the impact of aptamers with different sequences on the sensing of the same target rather than just focusing on their affinity to the target, which can help design aptasensors with suitable aptamers.

In recent years, some researchers have begun to pursue visualization of EC or PEC signals in order to meet the needs of miniaturization of instruments and detection on-site [108,109]. Zou et al. constructed an aptasensor for detection of DON in food using a small chip fabricated by laser etching to generate PEC and visual electrical signals simultaneously (Figure 4d) [93]. This aptasensor has the advantages of fast detection speed and good portability, and it exhibits good sensitivity and selectivity in detecting DON by the results of orthogonal experiments and fitting equations.

#### 2.2.4. Other Dual-Mode Aptamer Sensors

In recent years, various cutting-edge technologies combined with aptamer sensors have continuously emerged, promoting the innovation of detection technology for mycotoxins, bacteria, viruses, heavy metal ions, etc. [73,110,111].

**Figure 5:**
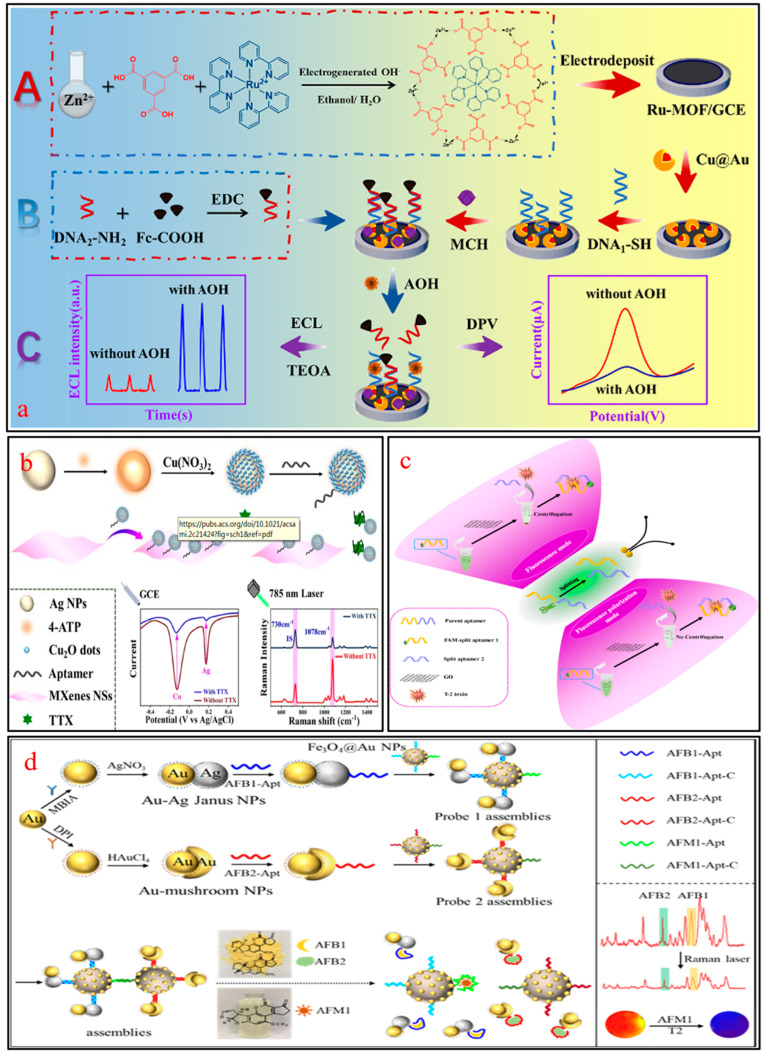
(**a**) ECL-EC aptasensor based on PoPD/Ru-Au for AOH detection; (**b**) Ag@Cu_2_O-engineered EC-SERS aptasensor for TTX detection, adapted with permission from Ref. [113]. 2024, Elsevier; (**c**) The detection of T-2 by the FL-FP split aptasensor; (**d**) Detection mechanism of three subtypes of AF.

Chemiluminescence (CL) connects the activity of molecules with luminescence through chemical methods, which helps to realize the detection of biomolecules. Jiang et al. combined electrochemiluminescence (ECL) with EC technology to design a dual-mode aptamer sensor based on the bifunctional nanomaterial PoPD/Ru-Au for the detection of alternariol (AOH) in fruits [112]. The complementarity of PoPD and Ru-Au effectively amplifies the ECL and EC signals, showing a wide response range (Figure 5a).

SERS technology is often used to construct multimodal biosensors because of its high sensitivity, freedom from labels, simplicity, and versatility. Recently, a study proposed a dual-mode SERS-CM aptasensor for ultra-sensitive detection of STX2 using Mn/Fe-MIL(53)@AuNSs as a CM probe, and modified AuNSs as a SERS probe [90]. Theoretical simulation and experimental verification have shown that Mn/Fe-MIL(53)@AuNSs have good peroxidase activity and high Raman response, and satisfactory recovery has been obtained in the detection of STX2 in milk. Yao et al. adopted Ag@Cu_2_O NPs as markers to design an anti-interference SERS-EC aptamer sensor to detect TTX in the range of 100 pg/mL–10 μg/mL. The EC and SERS signals were collected based on the specific peak intensities, with LODs of 31.6 pg/mL and 38.3 pg/mL, respectively (Figure 5b) [94].

The principle of fluorescence polarization (FP) immunoassay is that fluorescent substances absorb the energy of excitation light and emit polarized fluorescence in a single plane. Based on this, Zhu et al. designed an FL-FP split aptasensor for the detection of T-2 toxin in peanuts, as shown in Figure 5c [113]. Graphene oxide (GO) nanosheets serve as signal switches, and their presence or absence determines the on/off of FL and FP signals, which are used to determine T-2 toxin. The detection range of this sensor covers from 0.1 to 50 nM. In the detection of peanut samples, the recovery rates of FL and FP modes are 101.7–115.2% and 109.8–121.6%, respectively.

For the simultaneous detection of multiple toxins, Gao et al. designed a high-throughput biosensor based on SERS and magnetic relaxation switch (MRS) for the detection of three AF subtypes (i.e., AFB1, AFB2, and AFM1) [114]. As shown in Figure 5d, Au-Ag Janus NPs and Au mushroom NPs exhibit strong noninterfering SERS responses and were used as SERS nanolabels to distinguish AFB1 and AFB2. Meanwhile, Fe_3_O_4_@AuNP modified with the AFM1 aptamer was employed as an MRS probe to monitor AFM1. The designed dual-mode detection platform demonstrated good discrimination and amplification performance of SERS signals, as well as high transverse relaxation time, which achieved synchronous detection of AFB1, AFB2, and AFM1.

## 3. Aptamer Sensors for In-Field Detection

In-field testing differs from laboratory measurement in terms of purpose and method. In-field testing mainly uses qualitative analysis techniques to evaluate the presence of a specific target in a specific sample, while laboratory measurements usually use large instruments to quantitatively analyze the target. In terms of specific operations, for example, field testing equipment usually uses PCR and nucleic acid signal amplification technology, while laboratory measurements usually require the use of quantitative real-time PCR technology [115]. It could be argued that in-field testing is a prerequisite for laboratory measurement, and the two are jointly applied to the diagnosis and treatment of diseases.

**Figure 6:**
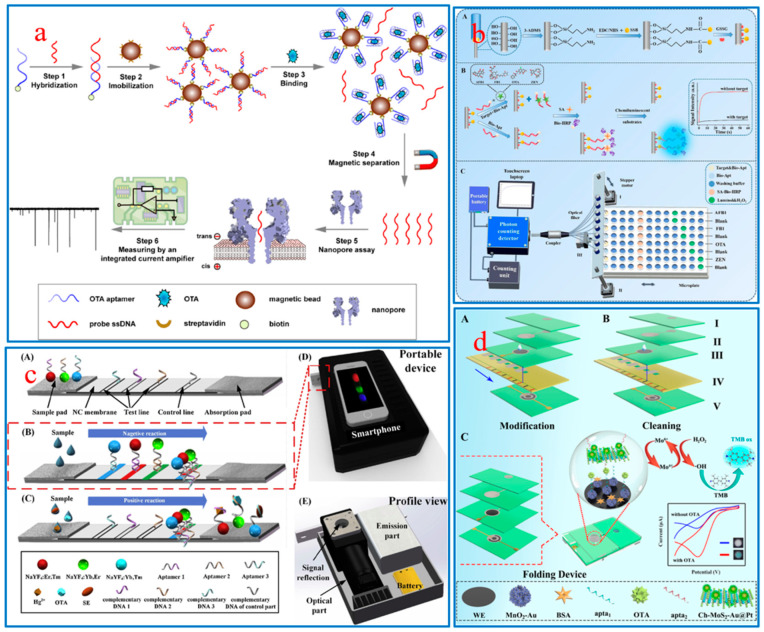
(**a**) Signal conversion strategy of the portable nanopore biosensor for OTA detection; (**b**) Portable CL-optical fiber aptasensor for analysis of multiple mycotoxins; (**c**) Structure and sensing principle of LFAA for simultaneous detection of multiple targets; (**d**) Scheme of modification (**A**), cleaning (**B**) and (**C**) sensing principle of EC-CM PAD.

In order to meet the requirements of real-time and rapid in-field detection, portable biosensors with small size, low cost, and simple operation (no need for professional operators) provide a feasible solution for early disease diagnosis, food quality detection, environmental pollution monitoring, etc. Biosensors can be combined with integrated signal readers to realize Plus-and-Play in detection devices, such as paper-based fluid devices [116], micro-fluidic chips [117], intelligent integrated devices [118], etc. At present, portable aptasensors have been applied in the detection of FBMTs, greatly reducing detection costs and time by directly converting target concentrations into visible signals. For example, a portable nanopore current amplifier was designed that relied on an immobilized aptamer-based nanopore signal conversion strategy for the detection of OTA [119]. As shown in Figure 6a, the preferential binding of OTA and aptamer releases single-stranded DNA (ssDNA) probes from the surface of magnetic beads, and then the released ssDNA is captured by the nanopore and converted into current signals. This sensing platform has realized the detection of OTA in corn, with an ultra-low detection line of 1.697 pmol/L. Some researchers have attempted to simultaneously identify and detect multiple mycotoxin targets in a single measurement in order to improve the detection efficiency of sensors. Jia et al. developed a CL-optical fiber aptasensor based on the specific recognition mechanism of ssDNA and aptamers that was used for synchronous determination of multiple FBMTs on-site [1]. The optical fiber biosensor consists of three parts: a signal display system consisting of a photon counting detector and a laptop, an eight-channel sensing fiber, and a sampling area (Figure 6b). This sensing system has shown excellent stability and selectivity in the synchronous detection of AFB1, FB1, OTA, and ZEN in infant cereals. In addition, Jin et al. developed an integrated portable device for lateral flow aptamer assay (LFAA) combined with a smartphone, which was used for the synchronous detection of multiple different types of targets [120]. As depicted in Figure 6c, by using multicolored UCNPs functionalized with aptamer as probes, the concentration of each target can be observed from the intensity of the corresponding color band through the competitive format. The biosensor synchronously detected three different types of target substances, OTA (small molecules), Ag^+^ (ions), and Salmonella (bacteria), in tap water within 30 min, and displayed the detection results on smartphone software. The advantage of dual-mode sensors comes mainly from their ability to provide two different signals, which can complement each other and ensure more accurate analysis results. However, dual-mode sensors typically require the collaboration of two different devices, which are bulky and have poor portability. In response to this challenge, the Feng group designed an EC-CM paper-based analytical device (PAD) to detect OTA by using wax printing and screen printing and Ch-MoS_2_-Au@Pt-apta_2_ as a sensing element [121]. The PAD integrates micro-fluidic chip, electrode, and dual-signal output components (Figure 6d). If there is OTA, the Ch-MoS_2_-Au@Pt-apta_2_ will specifically bind to it and catalyze the reduction of H_2_O_2_ to achieve EC signals. Then, the hydroxyl radicals (•OH) generated by catalysis flow toward the colorimetric region, promoting the oxidation of 3,3′,5,5′-tetramethylbenzidine for visual detection.

In recent years, research on the integration of aptamers into lab-on-a-chip (LOC) systems has received widespread attention [122,123]. The most prominent feature of LOC is its ability to perform laboratory procedures such as separation, mixing, washing, detection, and high-throughput signal transmission on a tiny chip [124]. Therefore, it is significant and challenging to logically and efficiently deploy these functional components on a chip substrate. To address these challenges, the development of the LOC-aptasensor device should concentrate on two key areas: First, it is essential to enhance the flexibility, conductivity, wear resistance, and mechanical resistance of the chip materials, while thoroughly exploring the theory of device biointerfaces in order to boost their biocompatibility. Second, it is essential to screen high-performance aptamers in reaction to the complexity of biological samples. Just as computer chip can miniaturize a computer, LOC can also miniaturize a laboratory, and the resulting integrated, automated, and portable LOC-aptasonser device is expected to provide valuable assistance for in-field detection of mycotoxins, bacteria, viruses, and more.

## 4. Conclusions and Outlook

This review provides an overview of the latest advances in aptamer-based biosensors for the detection, analysis, and evaluation of FBMTs, including basic research and on-site detection applications. With the inherent characteristics and integration effects of various scientific technologies, as well as the specific recognition of mycotoxins by aptamers, different types of aptasensors (including single-mode and dual-mode) have been developed. Compared to single-mode aptasensors, dual-mode aptasensors not only enhance detection sensitivity, reliability, accuracy, and efficiency, but also the flexibility in on-site detection by combining with small and micro devices such as paper-based fluid devices, micro-fluidic chips, and intelligent integrated devices. The present study indicates that some aptamer sensing platforms have reached the detection limits of fg/mL in detecting major FBMTs such as OTA, DON, and ZEN, demonstrating the effectiveness of aptamer-based sensing technology. Although some progress has been made in the research of aptamer biosensors, more progress is urgently needed to realize higher sensitivity, selectivity, and practicality.

First, there will be interference between two signals of dual-mode aptasensors, and the sensitivity of dual-mode sensing signals mainly depends on the stability and affinity of the aptamers. Notably, these two properties are often influenced by environmental factors such as temperature, pH, and impurity ions in a biological sample. Therefore, enhancing the specificity and affinity of aptamers through high-tech strategies (such as base mutations, skeleton modifications, and nucleic acid signal amplification technology) coupled with the necessary sample pre-processing can effectively reduce the interference of dual-mode signals.

Second, although aptamers and antibodies have the ability to specifically recognize biological targets, only a few aptamers and antibodies can be developed and utilized, limiting the application space of biosensors. Hence, the screening process should extend beyond aptamers and antibodies, with a broader range of identifying elements considered for various assay types, including peptides, synthetic polymers, molecular receptors, and other affinity agents.

Third, most aptasensors are proof-of-concept models that lack the ability for clinical practice, especially in the face of the complex actual samples. Therefore, continuous efforts to advance and refine aptasensor technology are crucial to increasing its practicality. To optimize the utility of aptamers in assay systems, it is crucial to investigate the impact of these environmental factors, as well as the actual composition of proteins and lipids in the sample, on aptamer-target binding. Researchers should make full use of computer technology to develop multidimensional structural simulation and prediction rather than just focusing on primary sequence analysis. In addition, simulating the docking of an aptamer and target and forming corresponding molecular dynamics models to more accurately evaluate the stability of aptamer-target complexes is also a future consideration.

Fourth, in response to the rising demand for miniaturization, user-friendliness, and intelligence in equipment, the development and commercialization of aptasensors for in-field detection is critically needed. Specifically, the smartphone, which has become a quintessential everyday device, can be transformed into an efficient portable analyzer if it is equipped with biological detection devices and corresponding mobile applications.

In summary, as a continuously developing technique, aptamer sensing is expected to provide more assistance for the detection of mycotoxins, bacteria, viruses, and heavy metal ions.

## Figures and Tables

**Table 1:** Summary of single-mode and dual-mode aptasensors for the detection of FBMTs.

	Method	Analyte	Actual Samples	Linear Range	LOD	Reference
Single-mode	CM	SEB	Milk	0.5 ng/mL–50 µg/mL	0.5 ng/mL	[50]
		MC-LR	Water	0.5 ng/mL–7.5 µg/mL	0.37 ng/mL	[76]
		STX	Scallop	0.044–11.241 ng/mL	43 pg/mL	[77]
		T-2	Wheat, corn	0.1–5000 ng/mL	57.8 pg/mL	[78]
		OTA	Liquor	0.05–50 ng/mL	9 pg/mL	[79]
	FL	TTX	Shellfish	0–0.149 µg/mL	0.979 ng/mL	[59]
		OTA		3.63–40.30 ng/mL	3.63 ng/mL	[55]
		OTA		0–10 ng/mL	1.9 ng/mL	[80]
		T-2	Beer	0.5–100 ng/mL	0.239 ng/mL	[81]
	EC	T-2		2 fg/mL–20 ng/mL	0.107 fg/mL	[63]
		OTA	Wine	0.1–20 ng/mL	30 pg/mL	[82]
		FB1	Maize	0.072 ng/mL–7.22 µg/mL	1.44 pg/mL	[83]
	SERS	PAT		0–70 ng/mL	0.038 ng/mL	[70]
		AFB1	Chinese herbs	0.01–100 ng/mL	0.36 pg/mL	[69]
		OTA	Chinese herbs	0.001–10 ng/mL	0.034 pg/mL	[69]
Dual-mode	CM-FL	OTA		40.38 fg/mL–40.38 pg/mL	13.79 fg/mL	[84]
		OTA		40.38 ng/mL–10.09 µg/mL	40.38 ng/mL	[85]
		OTA	Wheat	0.05–10 μg/kg	0.008 μg/kg	[86]
	SERS-FL	AFB1	Peanut	0.001–1000 ng/mL	0.45 pg/mL	[87]
		OTA	Beer	0.01–100 ng/mL	3.2 pg/mL	[88]
		OTA	Coffee	5–250 pg/mL	1.03 pg/mL	[89]
	SERS-CM	STX2	Milk	0.05–500 ng/mL	26 pg/mL	[90]
	EC-PEC	AFB1	Peanut, corn	0.02 pg/mL–100 ng/mL	9.3 fg/mL	[91]
		PAT	Juice	50 fg/mL–500 ng/mL	30 fg/mL	[92]
		DON	Beer	1 fg/mL–100 pg/mL	0.37 fg/mL	[93]
	EC-SERS	TTX	Fish	100 pg/mL–10 μg/mL	31.6 pg/mL	[94]

Abbreviations: MC-LR, Microcystin-LR; STX, Saxitoxin; OTA, Ochratoxin A; FB1, Fumonisin B1; AFB1, Aflatoxin B1; STX2, Shiga toxin type II.
